# “Immuno-Transient Receptor Potential Ion Channels”: The Role in Monocyte- and Macrophage-Mediated Inflammatory Responses

**DOI:** 10.3389/fimmu.2018.01273

**Published:** 2018-06-06

**Authors:** Giorgio Santoni, Maria Beatrice Morelli, Consuelo Amantini, Matteo Santoni, Massimo Nabissi, Oliviero Marinelli, Angela Santoni

**Affiliations:** ^1^Section of Experimental Medicine, School of Pharmacy, University of Camerino, Camerino, Italy; ^2^Department of Molecular Medicine, Sapienza University, Rome, Italy; ^3^School of Biosciences and Veterinary Medicine, University of Camerino, Camerino, Italy; ^4^Clinical Oncology Unit, Macerata Hospital, Macerata, Italy; ^5^Neuromed I.R.C.C.S. – Istituto Neurologico Mediterraneo, Pozzilli, Italy

**Keywords:** macrophages, transient receptor potential, macrophage polarization, migration, phagocytosis

## Abstract

Monocytes and macrophages play important roles in health and disease. They have a central role in protecting the host, as they clear pathogens and modulate other immune cell functions through the production of regulatory molecules. Their functions include immune surveillance, bacterial killing, tissue remodeling and repair, clearance of cell debris and more. Macrophages can have beneficial and detrimental effects on the outcome of several diseases depending on the microenvironment and the activation state of cells. Over the past few years, there has been an increasing interest in the expression and functions of ion channels, in particular of transient receptor potential (TRP) channel family in immune cells. The 30 members of mammalian TRP channels are subdivided into TRPC, TRPV, TRPM, TRPML, TRPP, and TRPA superfamily, and several members of TRP subfamily have been found to be functionally expressed in monocytes and macrophages. TRP are cation-selective channels that are weakly voltage-sensitive and diversely gated by temperature, mechanical force, electrophiles, ligands, and internal cues, such as membrane composition and pH, contributing to immune and inflammatory responses. The TRP channels play major roles in controlling several monocyte and macrophage functions such as phagocytosis, production of chemokines and cytokines, cell survival, polarization and so forth. In addition, they can also be potential therapeutic targets in a variety of inflammatory diseases. Thus, the goal of this review is to describe the role of TRP channels in the control of monocyte–macrophage functions in inflammatory and immune-mediated diseases.

## Introduction

Macrophages play a crucial role in defense and disease by triggering immune surveillance, bacterial killing, tissue remodeling, and tissue repair (1–4). Macrophages show beneficial or detrimental effects in different diseases depending on their cell activation state and the microenvironment where they are present (5).

In the last years, there has been an increasing interest in the expression and functions of transient receptor potential (TRP) ion channel family in myeloid cells.

On the basis of amino acid sequence homology, TRP channels are grouped into different subfamily, called canonical (TRPC), melastatin (TRPM), vanilloid (TRPV), ankyrin (TRPA), mucolipin (TRPML), and polycystin (TRPP) subfamily (6, 7). Structurally they have six transmembrane spanning domains (S1–S6) with a pore domain between the fifth (S5) and sixth (S6) segment and intracellular C and N termini (8–10). TRP channels conduct cations, are weakly voltage-sensitive and non-selective for calcium, with a permeability ratio to Na (*P*_Ca_/*P*_Na_) in a range between 0.3 and 10.

At present, TRP channel ligands are only partially known, although they function as multimodal signal integrators for exogenous ligands. The G protein-coupled receptors (G_q/11_; linked to PLCβ) and tyrosine kinase receptors (linked to PLCγ) potentiate the signaling and function of most TRP channels (11). Elements of phosphatidylinositol signaling pathway, in particular, PIP_2_, can regulate TRP channels (12). In addition, intracellular Ca^2+^ increases TRP activity and modulates all TRP channels. For detailed description of TRP channels, there are many excellent reviews (13–16).

Several members of TRP subfamily are expressed in monocytes and macrophages (M/MΦ) (17). In these cells, they can recognize exogenous signals, including damage-associated molecular pattern molecules from the environment (heat, acidity, and chemicals) and endogenous danger signals released during trauma/tissue injury (ATP, mechanical, osmotic stress, and uric acid). In addition, they sensitize the pattern recognition receptors expressed in myeloid cells to respond to pathogen-associated molecular patterns (PAMPs) (18).

Aim of this review is to describe the cellular functions mediated by different members of TRP channels in M/MΦ.

## Effects of TRP Channels on M/MΦ Survival and Proliferation

TRPM channels control the survival and proliferation of M/MΦ. In this regard, TRPM2 has been found to inhibit reactive oxygen species (ROS) generation in phagocytic cells and protect the mice from LPS-induced effects. LPS-treated *TRPM2*^(−/−)^ mice show an increased inflammatory response and reduced cell viability with respect to wild-type mice. In addition, TRPM2 channels damp NADPH oxidase-stimulated ROS generation by phagocytes, through the induction of plasma membrane depolarization (19). The other TRP family member, TRPM4, controls M/MΦ survival in sepsis (20). The knockout of the TRPM4 gene increases the mortality in a murine model of LPS-induced sepsis. The lack of TRPM4 affects peritoneal macrophage infiltrate and increases the monocyte number, and the release of IL-1β and TNFα cytokines. Macrophages from *TRPM4* knockout mice display reduced Ca^2+^ mobilization that inhibits the Akt pathway, and consequently macrophage survival, phagocytosis of bacteria (20).

**Figure 1:**
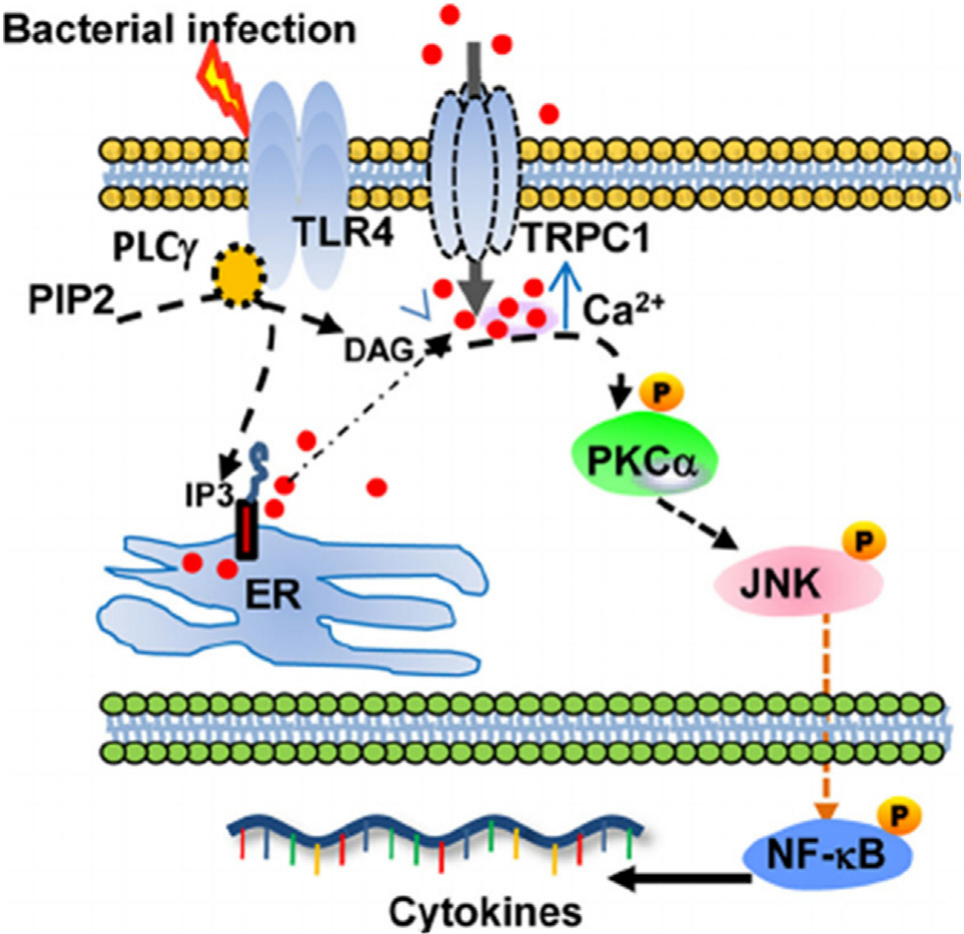
Schematic describing the proposed TRPC1/PKCα/JNK/NF-κB axis involved in the dysregulated pro-inflammatory response during bacterial infection. From Ref. (21) Copyright 2015 Molecular and Cellular Biology.

TRPC1 plays an important role in the protection from bacterial infection, through TLR4-TRPC1 activation of protein kinase (PK) Cα pathway (Figure 1) (21). Ca^2+^ entry, induced by TRPC1 channel, stimulates the production of pro-inflammatory cytokines in murine pneumocytes. The TLR4-dependent TRPC1 activation triggers Ca^2+^ depletion from endoplasmic reticulum (ER) store. After activation of PLC-γ, TRPC1 mediates Ca^2+^ entry and stimulates PKCα activity, which results in NF-κB/Jun kinase nuclear translocation and cytokine release leading to tissue destruction (21). The *TRPC1*^(−/−)^ mice show reduced survival, lung tissue damage, and systemic infection. Moreover, bone-marrow macrophages from *TRPC3*^(−/−)^ mice show reduction in basal Ca^2+^ influx, impaired TNFα-induced signal as compared to wild-type cells (22).

## TRP Channels and Inflammasome Activation in M/MΦ

The inflammasomes are multiprotein platforms that mediate pro-caspase-1 cleavage and promote cytokine maturation (e.g., IL-1β and IL-18), in response to microbial and non-microbial stimuli, by canonical and non-canonical mechanisms. The activation of non-canonical inflammasome is mediated by caspase-11 that triggers IL-1β, IL-18, and IL-1α release in a caspase-1-dependent and -independent manner. Caspase-11 also promotes pyroptosis, a form of genetically programmed cell death (23). TRPC1 represents a substrate for caspase-11. Defects in TRPC1 expression enhance caspase-1-independent IL-1β release or macrophage death. Thus, intraperitoneal LPS injection in *TRPC1*^(−/−)^ mice induces higher IL-1β secretion (24). Recently, in human U937 monocytes exposed to high glucose (HG) condition that induces the NLRP3-ASC inflammasome stimulation leading to caspase-1 activation and IL-1β and IL-18 secretion, TRPM2 regulates the thioredoxin-interacting protein-mediated triggering of NLRP3 inflammasome *via* interaction with the p47phox protein (25). In particular, TRPM2 activation and TRPM2-mediated Ca^2+^ influx represent the critical steps in NLRP3 activation. In response to HG, the reduction of TRPM2 expression reduces ROS generation and NADPH oxidase activity (25).

In phagocytes, the formation of crystals also induces oxidative stress that triggers NLRP3-mediated IL-1β secretion. Recently, Zhong et al. have demonstrated that liposomes are required for NLRP3 activation (26) and ROS-dependent TRPM2-mediated calcium influx (27). Infact, in macrophages from TRPM2 knockout mice, neither NLRP3 activation nor IL-1β production, is evidenced.

The NLRP3 inflammasome senses cell swelling and regulatory volume decrease (RVD), and the TRPV2 channel has been found to control volume regulation (18, 28). The reduction in extracellular osmolarity results in K(+)-dependent conformational change of the inactive NLRP3 inflammasome state followed by its activation, which is controlled by TRPV2 during RVD (28). Moreover, NLRP3-independent activation has been reported in human THP-1 macrophages (29). Apoptosis-associated speck-like protein containing a CARD domain (ASC) is required for the inflammatory processes. ASC bring NLRP proteins near to procaspase-1 into the inflammasome complex. Under hypotonic conditions, in TRPV2-dependent and independently by NLRP3, ASC forms specks that are unable to mediate pro-caspase-1 activation and pyroptosis. However, ASC speck formation leading to inflammasome and pro-caspase-1 cleavage is increased by interaction with NLRP3 (29).

## Contribution of TRP Channels to MΦ Polarization

Similar to the Th1/Th2 nomenclature (30, 31), in response to different cytokines or PAMPs, there are specialized and polarized M1 and M2 macrophages. Activated M1 macrophages are induced by IFNγ alone or by microbial stimuli (e.g., LPS) or cytokines (e.g., TNF and GM-CSF). IL-4 and IL-13 other than to be inhibitors of macrophage activation, can induce the alternative M2 phenotype of macrophages (30). Activated M2 macrophages include cells exposed to IL-4 or IL-13, immune complexes, IL-10, glucocorticoids, or hormones (32). M1 cells secrete high levels of IL-12 and IL-23 and exhibit low IL-10 production; they generate NO and ROS and produce IL-1β, TNF, IL-6; they participate in Th1-polarized responses and mediate increased resistance against intracellular parasites and tumors. In contrast, M2 macrophages secrete low levels of IL-12 and IL-23 and high levels IL-10. Low expression of IL-1β and caspase-1 and high levels of IL-1ra, and decoy type II receptor were found in M2 cells (33). M1 and M2 cells also have distinct chemokine and chemokine receptor repertoire (31). M2 cells cooperate with Th2 cells in promoting the killing of parasites (34); they are present in some tumors and stimulate tissue repair (35). Moreover, recently, the analysis of transcriptomes in human macrophages stimulated with different stimuli has revealed the presence of distinct stimulus-specific macrophage polarization program and a broader spectrum of macrophage activation states, other that M1 and M2 (36).

A number of evidences indicate that the TRP channels regulate macrophage differentiation. Thus, gastric inflammation and reduced bacterial colonization were observed in *Helicobacter pylori*-infected *TRPM2* knockout mice compared to controls (37). Loss of TRPM2 in *H. pylori*-infected macrophages triggers an increased production of inflammatory mediators and M1 polarization. Stimulation of TRPM2-deficient macrophages with *H. pylori* induces calcium overloading and increase of ERK1/2 and NADPH oxidase activities respect to wild type cells (37).

The expression and activity of TRPM7 are differentially regulated in bone-marrow derived murine M1 and M2 macrophages (38). Unlike M1 macrophages, in IL-4 stimulated M2 macrophages, higher TRPM7 current density (about 4.7-fold) was observed, whereas TRPM7 mRNA levels remain unchanged upon cell polarization. NS8593 and FTY720, two specific TRPM7 inhibitors, block IL-4- and M-CSF-induced macrophage proliferation and prevent M2 polarization. Inhibition of TRPM7 expression diminishes IL-4-induced arginase-1 mRNA levels and activity and completely inhibits the IL-4 or M-CSF mediated effects on TNF production in LPS-stimulated macrophages. In addition, TRPM7 inhibition decreases PI3K and ERK1/ERK2 phosphorylation levels and induces apoptosis in rat hepatic stellate cells (32, 39). In addition, adoptive transfer of macrophages from *TRPM8*-deficient mice, aggravates colitis, and IL-10 overexpression rescues M2 macrophage subpopulation. Thus, TNFα production in TRPM8-positive macrophages promotes the M1 macrophage phenotype and pro-inflammatory activity (40). Consequently, activation of TRPM8 channel in murine peritoneal macrophages triggers calcium transient currents in wild type but not *TRPM8*-deficient mice exhibiting defective phagocytosis and increased motility (40).

**Figure 2:**
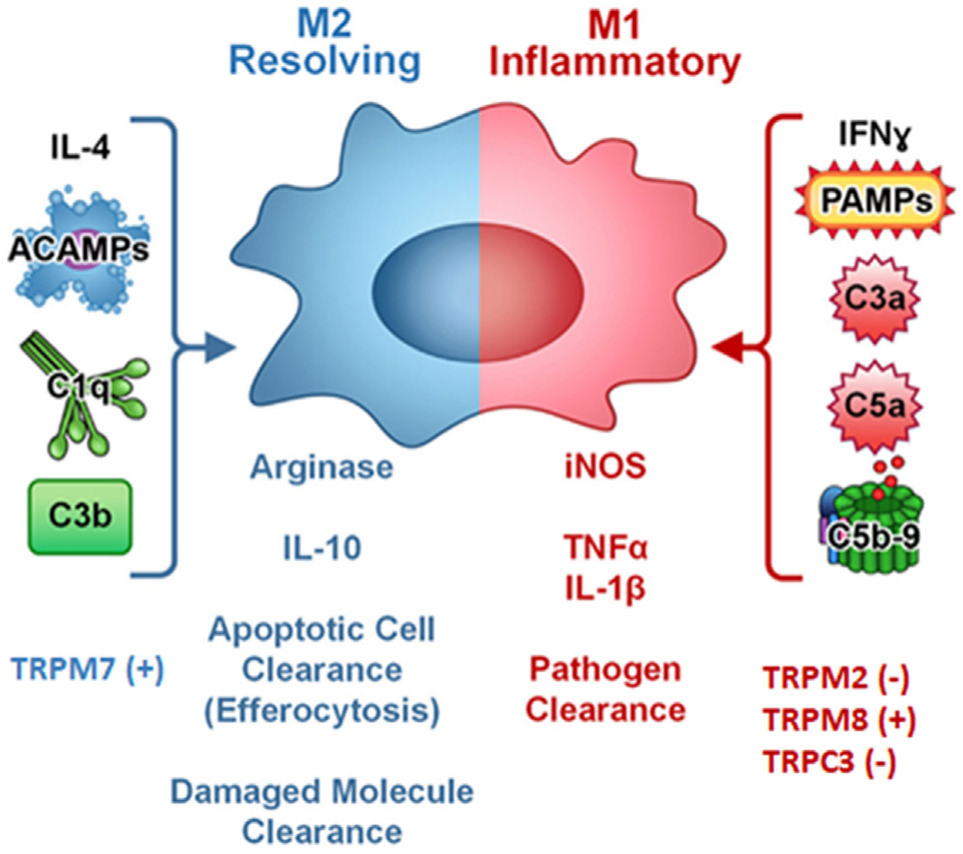
Complement components, cytokines, pathogen-associated molecular patterns (PAMPs), and TRP ion channels regulates macrophage polarization. Symbol (+) meaning stimulation, (−) meaning inhibition. Modified from (44) Copyright 2014 *Front Immunol*.

In addition, polarized macrophages from mice with specific *TRPC3* deficiency show an increased *in vitro* phagocytic function (22). A crosstalk between TRP channels and unfolded protein response (UPR) system regulating macrophage polarization was also evidenced (41, 42). Thus, in *Apoe*^(−/−)^ *TRPC3*^(−/−)^ mice, M1 but not M2 macrophages show diminished ER stress-mediated apoptosis is reported. The reduced susceptibility of *TRPC3*-deficient M1 macrophages to apoptosis induced by ER stress is associated with impaired UPR and down-regulation of pro-apoptotic molecules as calmodulin-dependent PK II (Figure 2) (22, 42–44).

## Role of TRP Channels in Adhesion and Migration in M/MΦ

Macrophage migration and infiltration is a multi-step process characterized by cell adhesion to different extracellular matrix (ECM) substrates, degradation of ECM proteins, topology and pericellular sense, intracellular transport, cell protrusion stabilization, and transmigration (45). In this regard, the organelles appointed to mediate these important functions are the podosomes. Recent studies have demonstrated that TRPV2 is localized in the podosome, and stimulation by fMLP further recruits TRPV2 to this compartment (46). Numerous signaling molecules including PI3K, Src, Cas, Pyk2, and Rho GTPases are associated with the podosome. TRPV2 may regulate Pyk2 activation, since *TRPV2* knockdown inhibits the phospho-Pyk2 expression in macrophages. Activation of Pyk2 by ionomycin leads to breakdown of the podosome. On the contrary, increase of podosome numbers upon Pyk2 blocking by using a dominant negative variant of PyK2, PRNK, was observed. Gelsolin-assembled actin filaments and gelsolin activity are required for podosome assembly. It can be suggested that TRPV2, by activating gelsolin, promotes the formation of podosome. In murine macrophages, TRPV2 also contributes to fMLP-induced Ca^2+^ entry and migration (47). Notably, translocation of TRPV2 to the membrane induced by fMLP stimulation, is completely abrogated by PI3K inhibition or by Gi/0 trimeric G protein, suggesting that trafficking of TRPV2 channel is PI-(3,4,5)-P3 (PIP3)-dependent (46, 47).

Overexpression of mouse TRPM7 channel results in focal adhesion (FA) formation, spreading, and adhesion by increasing Ca^2+^ levels. The transformation of FA into podosomes depends by a kinase-dependent TRPM7-mediated activation (48). Non-activated TRPM7 channel is not associated with the actomyosin protein in the cytoskeleton. Triggering with PLC agonists induces TRPM7-mediated Ca^2+^ influx and TRPM7 kinase activity. Autophosphorylation of TRPM7 protein promotes a conformational change in the channel structure that allows Ca^2+^-dependent myosin IIA association, myosin IIA heavy chain phosphorylation leading to myosin dissociation and cytoskeletal remodeling. Finally, silencing of TRPC6 by siRNA or treatment with SKF-96365, a TRP blocker induce cytoskeleton disruption in murine podocytes (49).

Cellular migration and contractility are regulated by cytoskeleton rearrangements, FA turnover and changes in Ca^2+^ flux. In this regard, a role for TRPM4 as regulator of FA/cytoskeleton dynamics, mechanotransduction, and adhesome has been reported (50). The mouse TRPM4 channel localizes at FAs, where it contributes to FA turnover and disassembly of lamellipodial actin cytoskeleton components. Moreover, TRPM4 by regulating FAK and Rac GTPase activities modulates cellular contractility and migration in M/MΦ (51).

TRPM2 is involved in chemokine production from M/MΦ (52). The expression of TRPML2 is negligible in resting macrophages, but its levels increase in response to TLR4, TRL7, and TLR8 stimulation. In activated macrophages, TRPML2 facilitates the fusion of recycling endosomes or plasma membrane, thus promoting secretion of specific chemokines and cytokines. Recent data (53) demonstrated that CCL2, CCL3, and CCL5 chemokines are reduced in *TRPML2*^(−/−)^ mice. Furthermore, *TRPML2* knockout mice display impaired recruitment of peripheral macrophages in response to intraperitoneal injection of either LPS or live bacteria (53). In human U937 monocyte cell line, CXCL8 production depends on TRPM2-mediated Ca^2+^ influx. Monocytes from *TRPM2* knockout mice exhibit reduced hydrogen peroxide-stimulated CXCL2 production (52). Activation of TRPM2 in human monocytes increases LPS-induced TNFα, IL-6, IL-8, and IL-10 production and phagocytosis *in vitro* (54).

The expression of TRPA1 mRNA in macrophages is upregulated in inflammatory bowel disease patients (55). In colitis, human TRPA1 channel activation exerts a mucosal protective role by reducing the expression of pro-inflammatory neuropeptides (SP, NKA, NKB, and VIP), cytokines (IL-1β, IFN, and TNF α/β), and of MCP-1 chemokine (55). Blocking of TRPA1 increases IL-10 levels and decreases TNF α secretion and TRPA1 siRNA normalizes monocyte IL-10 secretion (56).

## Contribution of TRP Channels in M/MΦ Phagocytosis

Macrophage phagocytosis of pathogens is essential function of innate immune responses and depends on a large repertoire of receptors capable to recognize different targets. Phagosome maturation requires endosomal pathway regulators, including the phosphoinositide lipids. Both, PtdIns(3,5)P2 and PIP3, are required for phagosome maturation. Inhibition of the lipid kinase that generates PtdIns(3,5)P2, PIKfyve, and phosphatidylinositol-5-phosphate [PtdIns(5)P] blocks phagosome-lysosome fusion and abrogates the phagosome degradative capability in RAW264.7 macrophages. PIKfyve inactivation disrupts membrane recycling by causing lysosome swelling and blocks phagosome and endosome maturation (57). In this regard, TRPML1 regulates phagosome biogenesis; both particle ingestion and lysosomal exocytosis are inhibited by TRPML1 blockers (56, 58). Instead, TRPML1 overexpression and TRPML1 agonist stimulation trigger lysosomal exocytosis and particle uptake. The particle binding stimulates lysosomal PI(3,5)P2 increase that triggers TRPML1-dependent lysosomal Ca^2+^ release, rapidly delivering TRPML1 translocation from lysosomal membranes to Lamp1^+^ nascent phagosomes (59, 60). PIKfyve and PtdIns(3,5)P2 trigger the TRPML1 channel to mediate phagosome–lysosome fusion. Genetic deletion of *TRPML1* gene hinders the acquisition of lysosomal markers in the phagosomes and reduces their bactericidal activity. Finally, cytosolic Ca^2+^ level increases during the TRPML1- and PIKfyve-dependent phagocytosis (57).

A role of TRPV2 in early phagocytosis was also demonstrated (61). The chemoattractant-elicited mobility, zymosan or complement-mediated particle binding, and phagocytosis are impaired in macrophages from *TRPV2* knockout mice. The TRPV2 recruitment to the nascent phagosome and plasma membrane depolarization increases PIP2 synthesis that triggers actin depolymerization indispensable for phagocytic receptor clustering (61). Moreover, recently recruitment of TRPV2 at cell surface, preferential localization in lipid rafts, and calcium influx upon *P. aeruginosa* infection have been reported. Furthermore, deregulated TRPV2-signaling in macrophages from cystic fibrosis is responsible for their defective phagocytosis and consequently chronic infection (62). Moreover, in RAW 264.7 macrophages, the TRPM8 activator icilin stimulates cation currents that result in macrophage membrane depolarization. It is intriguing to hypothesize that TRPM8 alters macrophage efferocytosis by inducing actin depolymerization and indirectly influences Ca^2+^-dependent macrophage survival or apoptosis by reducing the driving force for Ca^2+^-mediated positive feedback on other Ca^2+^ permeable channels (63).

Finally, Riazanski et al. have demonstrated that TRPC6 channel translocation into phagosomal membrane increases phagosomal functions. TRPC6 channel restores microbicidal function in compromised alveolar macrophages from cystic fibrosis patients (64).

Collectively, these findings indicate that TRP expression sensitizes M/MΦ to recognize phagocyte bacteria, and defective TRP channel expression and function lead to inefficient bacterial killing. Thus, TRPV4 mediates LPS-stimulated murine macrophage phagocytosis of *Escherichia coli in vitro* and opsonized particles *in vitro* and *in vivo* in mice model (65). Intracellular Ca^2+^ is a second messenger in TLR4-dependent recognition and signaling (65). In this regard, Ca^2+^-depletion in *TRPV2-*deficient mice challenged with *Listeria monocytogenes* induces accelerated mortality and greater bacterial organ load (61).

TRPM2 is required for bacterial clearance in *E. coli* sepsis. Thus, during polymicrobial sepsis, macrophages from *TRPM2* knockout mice show inefficient bacterial killing and increased infection and death. Disruption of TRPM2 affects phagolysosomal acidification, impairs the phagosome-lysosome fusion, impedes the phagosome maturation, and increases intracellular Ca^2+^-facilitated phagosome maturation in *TRPM2*^(−/−)^ macrophages (66). *TRPM2*^(−/−)^ mice are also extremely susceptible to *Listeria monocytogenes* infection and exhibit a defective innate immune response (19, 67). Similarly, the catalase from *Francinella tularensis* restricts ROS generation by hindering TRPM2-dependent Ca^2+^ entry in murine macrophages (68). In addition, TRPM2 disruption reduces heme oxygenase-1 expression and increases bacterial-induced macrophage infiltration. Pretreatment of macrophages from *TRPM2* knockout mice, with heme oxygenase-1 inducer, reduces bacterial burden (69). Finally, suppression of macrophage activation through inhibition of the TRPC1 activity has been evidenced in parasites-(helminths) induced diseases (70).

## Conclusion

Several evidences suggest the involvement of ion channels, in particular of TRP cation channel superfamily, in the pathogenesis of immune-mediated chronic inflammatory diseases. In this regard, the study of TRP channel functional expression in the M1/M2 macrophage polarization is an interesting research field to better understand how ion channels might participate in the generation of endogenous signaling capable of modifying macrophage polarization and differentiation, in the view to maintain health or to induce diseases. Crosstalk between inflammatory receptors and ion channels belonging to the TRP channel superfamily and the specific signaling pathway activated upon protein to protein interaction have been only partially elucidated and the contribution of a single TRP channel in the inflammatory response is still lacking. Further studies, both *in vitro* and *in vivo* aimed at uncovering the direct impact of different members of TRP subfamily in inflammatory processes are required. Thus, there is the need in the next future to explore and fully characterize the monocyte and macrophage expression of specific pattern of TRP channels and their signaling pathways activated in different immune-mediated diseases in order to identify new molecular targets for therapy of these inflammatory conditions.

## Author Contributions

GS supervised the work and wrote the manuscript. CA and MM contributed to the preparation of the subchapters about TRP channels and macrophage phagocytosis and migration. MN and OM cooperated in the preparation of the subchapters about TRP channels and macrophage survival and polarization. MS collaborated in the drafting of the introduction and conclusion. AS provided critical revision of the manuscript.

## Conflict of Interest Statement

The authors declare that the research was conducted in the absence of any commercial or financial relationships that could be construed as a potential conflict of interest.
